# Intravital Imaging of Inflammatory Response in Liver Disease

**DOI:** 10.3389/fcell.2022.922041

**Published:** 2022-06-28

**Authors:** Yang Wang, Jing Wang

**Affiliations:** Shanghai Institute of Immunology, Department of Immunology and Microbiology, Shanghai Jiao Tong University School of Medicine, Shanghai, China

**Keywords:** intravital imaging, innate immune system, adaptive immune system, recruitment, zonation

## Abstract

The healthy liver requires a strictly controlled crosstalk between immune and nonimmune cells to maintain its function and homeostasis. A well-conditioned immune system can effectively recognize and clear noxious stimuli by a self-limited, small-scale inflammatory response. This regulated inflammatory process enables the liver to cope with daily microbial exposure and metabolic stress, which is beneficial for hepatic self-renewal and tissue remodeling. However, the failure to clear noxious stimuli or dysregulation of immune response can lead to uncontrolled liver inflammation, liver dysfunction, and severe liver disease. Numerous highly dynamic circulating immune cells and sessile resident immune and parenchymal cells interact and communicate with each other in an incredibly complex way to regulate the inflammatory response in both healthy and diseased liver. Intravital imaging is a powerful tool to visualize individual cells *in vivo* and has been widely used for dissecting the behavior and interactions between various cell types in the complex architecture of the liver. Here, we summarize some new findings obtained with the use of intravital imaging, which enhances our understanding of the complexity of immune cell behavior, cell–cell interaction, and spatial organization during the physiological and pathological liver inflammatory response.

## Introduction

The liver is the main organ maintaining the homeostasis of substance and energy metabolism. It utilizes oxygen to uptake, metabolize, and store or eliminate biomolecules carried by blood, such as vitamins, hormones, ammonium, fatty acids, amino acids, glucose, and various waste products. This highly vascularized organ receives both arterial and portal venous blood, rapidly exchanges energy and matter in the liver sinusoid, and functions as a physiological bridge between gut-derived molecules and systemic circulation (Trefts et al., 2017). Venous blood from the intestine and spleen entering the liver *via* the portal vein also brings large amounts of foreign molecules and bacterial products. Consequently, the immune system of the liver is constantly exposed to endogenous oxidative stress caused by metabolic activity and numerous exogenous pathogens. In a healthy liver, the immune system responds to dangers caused by these stressors and eliminates them by a timely and tightly controlled inflammatory response (Robinson et al., 2016; Ahmed et al., 2021). The inflammatory response in physiological states can be spontaneously resolved and is essential for liver development, regeneration, and maintenance of normal function. Due to the self-limited characteristics of the liver immune system, it can automatically clear dead cells and dangerous signaling molecules, regaining immune homeostasis even when severe acute infection or injury is encountered. However, an uncontrolled inflammatory response can cause severe damage to the liver. The common feature of liver disease is excessive or persistent inflammation induced by diverse triggers, such as chronic infection, tissue damage, excessive consumption of alcohol or fat, and neoplasia. Under these conditions, persistent pathological inflammation can lead to fibrosis and even irreversible liver cirrhosis. Targeting immune cells represents a promising therapeutic strategy for the treatment of liver diseases (Robinson et al., 2016; Koyama and Brenner, 2017).

Advanced techniques such as multiplex flow cytometry, immunofluorescence, single-cell sequencing, and spatial transcriptomics enabled a better understanding of the relationship between liver inflammation and disease, particularly in nonalcoholic steatohepatitis (NASH) (Ramachandran et al., 2019). However, these approaches only provide static and *ex vivo* information and do not allow evaluating the dynamic behavior and interaction patterns of immune cells in the inflammatory response in the unique anatomic structure and microenvironment of the liver. Intravital microscopy (IVM) is a powerful tool for direct observation of cellular events within the intact organ of living animals. IVM with high spatiotemporal resolution can visualize immune cell behavior at single-cell or subcellular and millisecond levels (Germain et al., 2012). In the past decade, IVM had been used to study various pathophysiological processes in the liver, including blood flow, material transport, cellular behavior, tissue repair, and inflammatory response (Goetz et al., 2011; Thorling et al., 2014; Dasari et al., 2015; Marques et al., 2015; Reif et al., 2017; Matsumoto et al., 2018). Rapid improvement in microscopy techniques has permitted a comprehensive understanding of the cellular behavior and cell–cell interaction in hepatic inflammation, a process that underlies many liver diseases. In this review, we will focus on how IVM of the liver can improve our understanding of inflammatory responses in physiological and pathological states and discuss how recent technological advances and new findings can promote further research on inflammation-related liver diseases.

## IVM to Understand the Immune Composition and Microenvironment in the Healthy Liver

**FIGURE 1:**
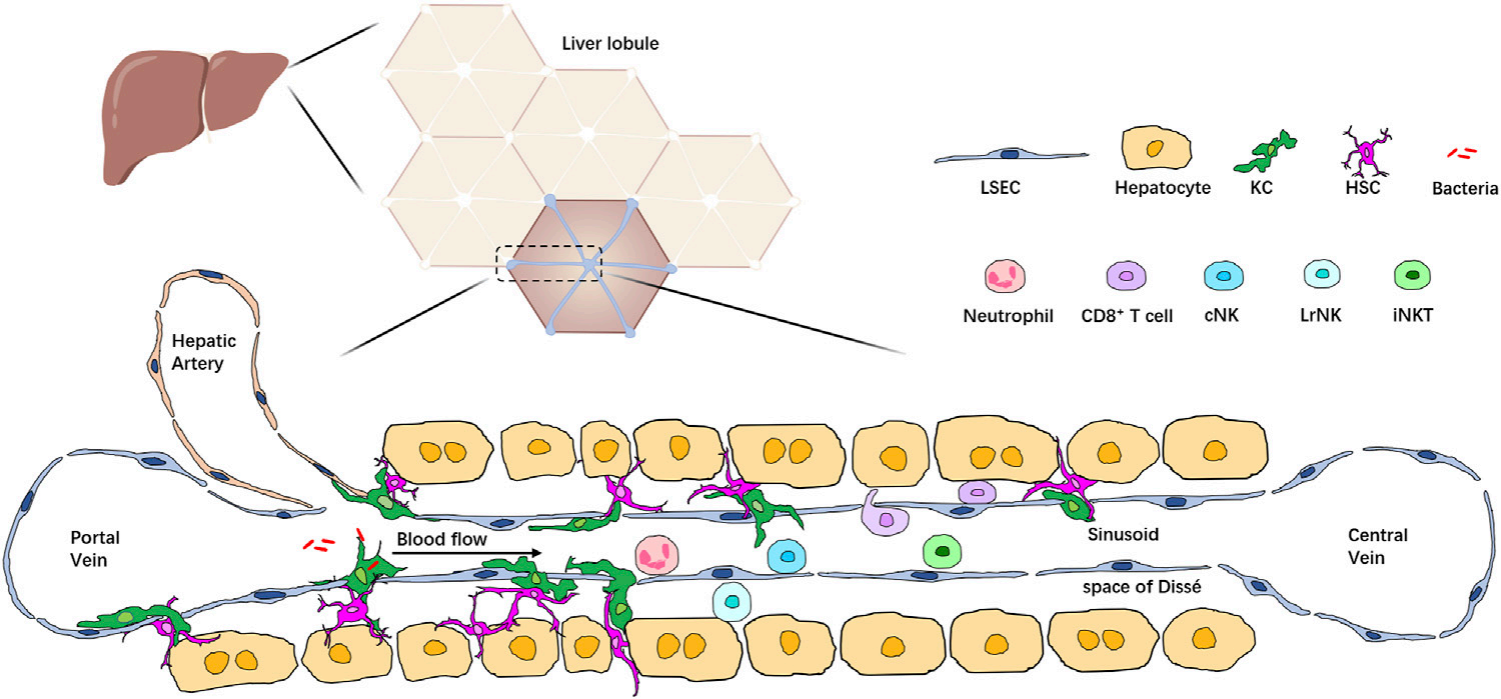
Localization of multiple types of immune cells in the liver lobule. The liver comprises hexagonal units called lobules. Blood from the portal vein and hepatic artery flows through liver sinusoids to the central vein. Hepatocytes are arranged around the sinusoid and form the space of Dissé. KCs are located in the space of Dissé and in close contact with HSCs, hepatocytes, and LSECs, allowing them to filter bacteria passing through the sinusoid. Immune cells such as neutrophils, T cells, iNKT cells, and conventional NK cells (cNK) are enriched in the hepatic sinusoids, patrolling the whole liver, whereas liver resident NK cells (LrNK) are located in the space of Dissé.

The healthy liver is a complex organ with a unique immunological microenvironment created by a diverse repertoire of immune and non-hematopoietic cell populations. Together, these cells form the basis of liver inflammatory response. In the liver, a vast array of cell types, including hepatocytes, hepatic stellate cells (HSCs), biliary cells, liver sinusoidal endothelial cells (LSECs), and immune cells, are arranged in a highly integrated manner into hexagonal units called lobules. Blood from the hepatic artery and portal vein mixes in the liver sinusoid and enters at the periphery of the highly vascularized lobule. Immune cells make up about 10% of all cell types in the liver; they can be divided into two groups: liver resident immune cells and circulating immune cells. Liver resident immune cells populate the liver sinusoid and the subendothelial compartment between hepatocytes and LSECs, known as the space of Dissé. Resident immune cells are found in the liver independent of the circulation (Knolle and Wohlleber, 2016; Ahmed et al., 2021). Liver resident immune cells, together with the circulating immune cells in the blood within liver sinusoids, form an extensive immune system network for effective immune surveillance and detecting and removing pathogens, toxic metabolites, and malignant cells (Jenne and Kubes, 2013). Recent advancements in liver IVM and the development of novel reporter tools and fluorochrome-conjugated probes allow us to monitor most immune cells at a single-cell level and have improved our understanding of the immune microenvironment in the liver (Li and Zeng, 2020) (Figure 1).

### Kupffer Cell

Dwelling in the sinusoid and the space of Dissé, Kupffer cells (KCs) strongly interact with HSCs, LSECs, and hepatocytes (Bonnardel et al., 2019). KCs are liver-specific macrophages representing 80–90% of all tissue macrophages in the human body and are considered the most powerful phagocyte in the liver due to their high phagocytic activity (Jenne and Kubes, 2013). Liver IVM revealed that KCs extend the portion of their cell body in the sinusoid lumen, forming lamellipodium-like structures that are continually scanning and monitoring the blood (Surewaard et al., 2016). Within minutes, KCs can capture most of the pathogens present in the blood, including Gram-positive and Gram-negative bacteria or even *Plasmodium berghei* sporozoites (Wardlaw and Howard, 1959; Frevert et al., 2005; Surewaard et al., 2016; Zeng et al., 2016), demonstrating the pivotal function of KCs in preventing the systemic spread of blood-borne pathogens. Mechanistically, KCs express a set of scavenger receptors, such as TLRs, complement receptors, and antibody receptors, that allow them to efficiently recognize, bind, and internalize pathogens (Zeng et al., 2018). IVM-utilizing fluorescent dyes OxyBURST and pHrodo visualized that the killing process of bacteria captured by KCs in phagolysosome involves acidification and production of reactive oxygen species (ROS) (Surewaard and Kubes, 2017).

Like most tissue-resident macrophages, KCs develop in the embryo from hematopoietic stem cells. This population is seeded in the liver during waves of embryonic hematopoiesis and subsequently self-renews, maintaining their number independently of replenishment by bone marrow–derived monocytes during adulthood (Ginhoux and Guilliams, 2016; Liu et al., 2019). The ability of KCs to maintain homeostasis can be explained by the “niche” model (Guilliams et al., 2020; Cheng et al., 2021). This fundamental concept contends that KCs in the liver are strongly adapted to the site of their residence. KCs and their niches are mutually beneficial. The niche provides a physical foundation for their accommodation, nutrients for their self-maintenance, and critical signals to imprint their tissue-specific identity. In turn, KCs are programmed by niche signals to participate in the essential functions of the liver (Zhou et al., 2018; Guilliams et al., 2020). Several studies showed that circulating monocytes can also replenish the KC pool in adult mice after depleting KCs by irradiation, clodronate-loaded liposomes, or the introduction of the KC-specific Clec4f-driven DTR gene (Beattie et al., 2016; David et al., 2016; Scott et al., 2016). These recruited monocytes gradually acquire KC identity, function, and self-maintenance capacity after they occupy the KC residence site in the liver, suggesting the existence of KC niches. Using IVM, Bonnardel et al. found that circulating monocytes were arrested in one specific spot after KC depletion and, in a manner similar to KCs, began to elongate and extend their pseudopods through LSECs to the parenchyma (Bonnardel et al., 2019). This study elaborately illustrated the continuous close contact of KCs with HSCs, LSECs, and parenchymal cells in KC niches. Moreover, it revealed distinct roles of these nonimmune cells in KC niche composition, especially the potential important function of LSEC-mediated DLL4-Notch signaling and HSC-associated BMPs in acquiring KC-specific identity (Sakai et al., 2019). Our recent work has further demonstrated that BMP9 and BMP10 control the identity and self-renewal of KCs through the ALK1-Smad4–dependent pathway (Zhao et al., 2022). Ablation of ALK1 or Smad4 in KCs resulted in a dramatic reduction in the expression of KC-specific transcription factors Id1 and Id3, indicating the loss of KC identity. Though these cells retain the CD64^+^F4/80^+^ macrophage phenotype, they do not express KC-specific surface markers such as Clec4F, Tim4, and VSIG4. The Alk1-deficient KCs also showed decreased proliferation, which is required to maintain KC homeostasis, and were replenished by circulating monocytes recruited into the liver. IVM observation of these “identity-deprived” KCs showed a significant reduction in bacterial capture capacity, leading to increased susceptibility to *Listeria monocytogenes* in Alk1/Smad4-deficient mice. These experiments highlight the ability of KC niches to imprint the identity and profoundly affect the functions of residing cells.

### Liver Capsule Macrophage

It has long been recognized that the liver resident macrophage population may be heterogeneous (Armbrust and Ramadori, 1996). However, it was only in 2017 that a distinct subset of these cells in the hepatic capsule was identified using intravital imaging (Sierro et al., 2017) and was initially classified as dendritic cells (DCs) (Prickett et al., 1988; David et al., 2016). Unlike KCs that occupy the liver sinusoid, liver capsule macrophages (LCMs) are located right underneath the liver capsule and express surface markers CX3CR1 and MHCII, but not Clec4F and Tim4 (Sierro et al., 2017; Bonnardel et al., 2019). In addition, LCMs lack self-renewal capacity and require replenishment by blood monocytes to maintain their number. LCMs exhibit “sampling” behavior at the interface between the peritoneal cavity and liver parenchyma, sensing peritoneal bacteria and promoting neutrophil recruitment to prevent bacterial invasion through the liver capsule. The identification of LCMs highlights the critical role of different tissue localization and microenvironment in the functional specialization of macrophages in the liver.

### Neutrophils

Neutrophils are typically the first leukocytes to be recruited to the inflammatory site and are capable of eliminating pathogens by multiple mechanisms (Mantovani et al., 2011; Phillipson and Kubes, 2011; Kolaczkowska and Kubes, 2013). These cells develop in the bone marrow and have a short lifespan after their release into the circulation (Ng et al., 2019). The liver, together with the lung and spleen, is considered a reservoir of mature neutrophils, also known as the “marginated pool,” that is retained in these organs in a steady state (Casanova-Acebes et al., 2018; Ballesteros et al., 2020). This population of neutrophils, also referred to as the “intravascular pool”, may support tissue regulatory functions and provide immune protection and surveillance under steady state (Casanova-Acebes et al., 2018; De Filippo and Rankin, 2020). It should be noted that the discovery of marginated neutrophils does not imply the presence of tissue-resident neutrophils since they were shown to be circulating cells retained for a prolonged time in the organ rather than developing independently from bone marrow neutrophils (Summers et al., 2010). IVM visualization of the neutrophil behavior in the healthy mouse liver showed that most of them flow rapidly with the bloodstream through the sinusoids; however, a small part of neutrophils was found to adhere to the LSECs and remain stationary or exhibit a crawling movement pattern in the steady state. These cells may represent the marginated population of neutrophils in the liver (McAvoy et al., 2011).

### Natural Killer Cells

Natural killer (NK) cells normally are rare in most tissues. They represent only a small fraction of circulating lymphocytes but account for up to 50% of intrahepatic lymphocytes in humans and 10% in mice (Male, 2017; Zhou et al., 2020). These innate lymphocytes play a key role in immune surveillance and are the main participants in the inflammatory response against abnormal cells during infection or oncogenesis. A balance of activating and inhibitory receptors on the NK cell surface allows NK cells to recognize the absence of normal self-molecules and kill the target by releasing cytotoxic granules, such as perforin and granzyme, and cytokines, such as IFN-γ (Zhou et al., 2020). Flow cytometric analysis identified two distinct subsets of NK cells in the liver: DX5^+^CD49a^−^ circulating conventional NK cells (cNK) and DX5^−^CD49a^+^ liver resident NK cells (LrNK) (Peng et al., 2013). Moreover, in contrast to cNK cells, LrNK cells develop from hematopoietic progenitor cells in the liver but not from the bone marrow. Moreover, LrNKs require different transcription factors, including T-bet, while cNK cells require the Eomes transcription factor (Gordon et al., 2012; Constantinides et al., 2014; Klose et al., 2014; Mackay et al., 2016; Zhang et al., 2016; Bai et al., 2021). Recent studies have revealed new, unexpected functions of LrNK cells, including their innate memory capability (Wang et al., 2019; Sun et al., 2012; O'Leary et al., 2006) and regulation of adaptive immune priming (Zhou et al., 2019). However, IVM studies on the dynamic behavior of cNK cells and LrNK cells are still missing due to lack of specific probes or reporter strains to mark these cells.

### Invariant Natural Killer T Cells

The liver contains the largest population of NKT cells in the body. These lymphocytes co-express NK receptors, such as NK1.1, and T cell antigen receptors (TCRs), representing up to 30% of lymphocytes in the liver. Most liver NKT cells express a semi-invariant TCR containing the Vα14-Jα18 chain (in mice) and Vα24-Jα15 TCRα chain (in humans) and a limited repertoire of TCRβ chains (Kronenberg and Gapin, 2002; Bendelac et al., 2007). These invariant natural killer T cells (iNKT cells) recognize a limited number of glycolipid antigens presented by CD1d-positive cells (Matsuda et al., 2000). IVM using CXCR6-GFP transgenic mice provided a strategy to visualize the behavior of iNKT cells in the liver (Geissmann et al., 2005; Heymann et al., 2015a). About 80% of GFP^hi^ cells in the liver are CD1d-reactive iNKT cells. These cells attach to the sinusoidal wall and continuously crawl at a slow speed (about 20 μm/min), within the sinusoids under basal conditions. The direction of this crawling motion is independent of the location of central veins or the direction of blood flow, suggesting that these cells are patrolling liver sinusoids and autonomously scanning for their cognate ligand. Activation of iNKT cells by glycolipid α-galactosyl ceramide (α-GalCer) presented by CD1d-positive cells stops the movement of iNKT cells immediately. The activated iNKT cells perform their effector role by secreting significant amounts of cytokines or exerting cytotoxic functions directly when encountering pathogen invasion or malignant cells (Kawano et al., 1998; Lee et al., 2015; Crosby and Kronenberg, 2018).

### T Cell Surveillance and Tolerance in the Liver

The liver was thought to be a unique organ representing an environment of immunotolerance for lymphocytes, possibly preventing excessive immune response to harmless routine exposure to food antigens or gut microbiota (Crispe, 2003). Although the highly permeable architecture of liver sinusoids permits the contact of naïve T cells with antigen-presenting cells (Warren et al., 2006), the generation of cytotoxic T cells in the liver was found to be inefficient and was largely attributed to the immunosuppression properties of hepatic antigen-presenting cells (APCs) including hepatocytes, LSECs, KCs, and HSCs (Wong et al., 2015). Normally, these cells in the liver maintain a low expression of costimulatory molecules and constitutively express anti-inflammatory cytokines such as IL-10 to suppress the activation of adaptive immune response (Knolle et al., 1998; Wahl et al., 2008). Moreover, LSECs and KCs express programmed death-ligand 1 (PD-L1) which limits the killing function of T cells after antigen presentation (Limmer et al., 2005; Pose et al., 2021; Triantafyllou et al., 2021). Consequently, naïve T cells activated by hepatic APCs fail to differentiate into robust effector T cells, but this immune restraint in the liver may also be the major reason for susceptibility to chronic hepatitis B and C viral infections (Guidotti and Chisari, 2006; Kennedy et al., 2017). Intravital imaging of the dynamics of naïve CD8^+^ T cells showed that the liver can support the differentiation of CD8^+^ T cells into effector cells when primed by KCs but not hepatocytes (Bénéchet et al., 2019). This finding demonstrated that the presentation of liver-expressed antigens does not always result in CD8^+^ T cell tolerance. The failure of differentiation into cytotoxic T cells was usually associated with continuous exposure to a high level of antigen in the liver, representing another mechanism of CD8^+^ T cell tolerance, known as exhaustion (Tay et al., 2014).

Taking advantage of the slow blood flow and fenestrated endothelium, circulating effector CD8^+^ T cells can also patrol and survey the liver. Using intravital imaging, Guidotti et al. established that effector CD8^+^ T cells can arrest within the sinusoid by docking onto platelets. Docked cells adhere to sinusoidal hyaluronan *via* CD44 and crawl along the sinusoids, scanning sub-sinusoidal hepatocytes by extending cytoplasmic protrusions through endothelial fenestrae (Guidotti et al., 2015). Recognition of cognate antigens permits effector T cells to migrate into the space of Dissé and trigger effector functions to kill the infected or malignant hepatocytes. Thus, IVM observations provided invaluable information on the dynamic behavior of naïve and effector T cells in the unique architecture and microenvironment of the liver and pointed to possible strategies to control adaptive immune response during chronic hepatitis B and C virus infections.

### Imaging the Immune Zonation in the Liver

Due to the widespread exchange of materials between blood and hepatocytes in the hepatic lobule, gradients of oxygen, nutrients, and noxious products are formed along the length of the sinusoid. Hepatic parenchymal cells also show partitioning of functions based on their location, a phenomenon known as “metabolic zonation” (Trefts et al., 2017; Cunningham and Porat-Shliom, 2021). Subsequent studies revealed that HSCs and LSECs also exhibit similar broad functional zonation patterns along the lobular radial axis (Halpern et al., 2018; Dobie et al., 2019). The spatial distribution of immune cells in the liver was also shown to be asymmetric (Gola et al., 2021). Kupffer cells and NKT cells are enriched in the periportal region; this spatial polarization of immune cells is termed “immune zonation.” Sustained MYD88-dependent signaling induced by commensal bacteria in LSECs controls the immune zonation of KCs by forming chemokine CXCL9 gradients. The zonal distribution of KCs was, in turn, shown to be more efficient in protecting against systemic bacterial dissemination than a uniform distribution (Gola et al., 2021). Immune zonation within the liver is an evolving area of research and still faces many emerging challenges. Although several studies utilizing advanced spatial transcriptomic and proteogenomic techniques have provided a cellular atlas of the hepatic immune microenvironment, whether the gene expression profiles of KCs are also zonated remains to be answered (MacParland et al., 2018; Andrews et al., 2022; Guilliams et al., 2022). Moreover, whether the other types of immune cells also exhibit similar zonation still needs to be confirmed. Finally, given the central role of KCs in liver inflammation (Koyama and Brenner, 2017), the question of how the immune zonation of KCs affects the inflammatory response occurring in the portal zone versus the central zone warrants further investigation.

## IVM to Understand Immune Cell Behavior During Inflammation

Inflammation is a highly dynamic process that relies on the coordination of the activity of multiple immune cell types. IVM has been extensively utilized to visualize cellular behavior during the inflammatory response. These observations provide direct and compelling evidence that facilitates our understanding of how immune cells act at different stages of inflammation.

### Imaging the Recruitment Cascade of Leukocytes

**FIGURE 2:**
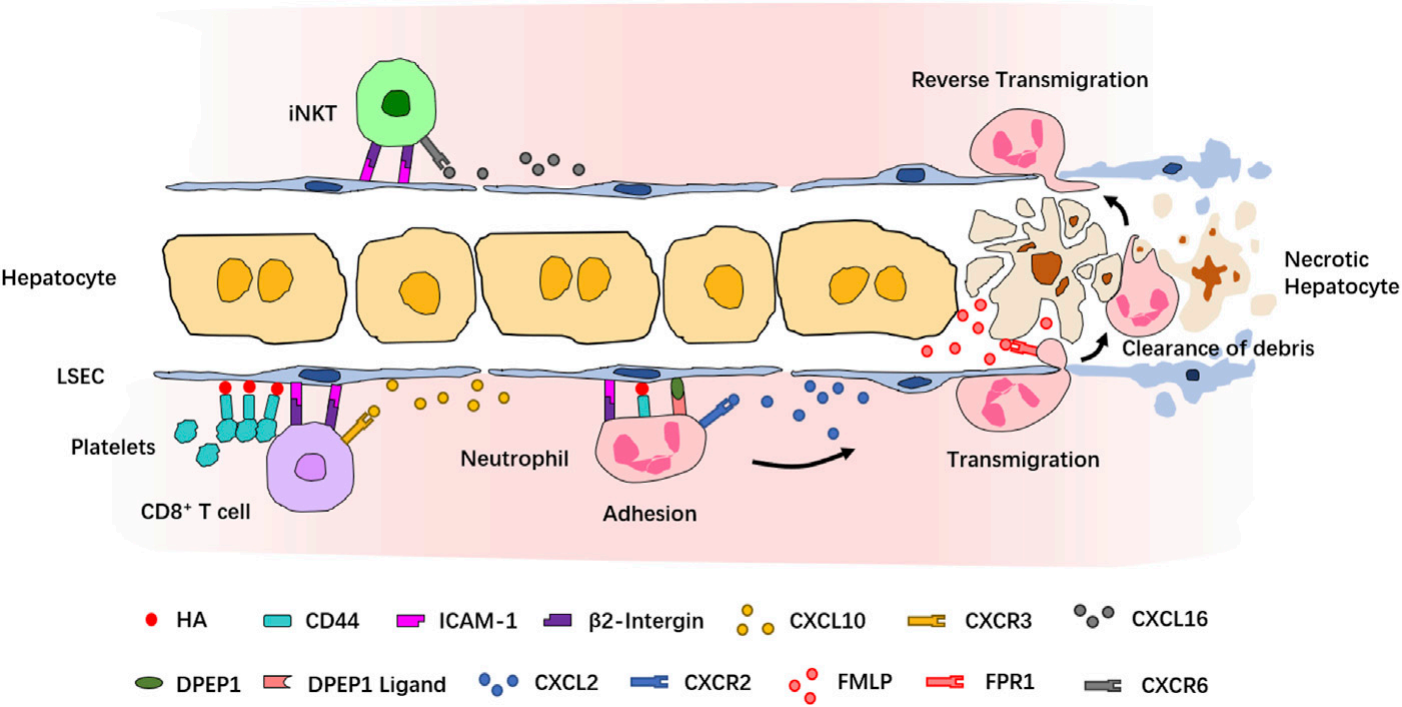
Recruitment cascade of leukocytes during inflammation revealed by IVM. Neutrophils are the first population recruited into the site of inflammation. In sterile injury-induced liver inflammatory response, neutrophils are attracted by the chemokine CXCL2 and adhere to LSECs *via* a series of adhesion molecules. Formyl-peptide released by necrotic hepatocytes mediates neutrophil transmigration to reach the necrotic cells and clear the debris of dead cells. After the clearance, neutrophils return to the sinusoids *via* reverse transmigration. CD8^+^ T cells and iNKT cells are attracted by, respectively, CXCL10 and CXCL16 and adhere to LSECs *via* similar mechanisms. Platelets also contribute to the retention of CD8^+^ T cells.

A functioning immune system responds to inflammatory stimuli by recruiting circulating immune cells into the site of inflammation. A highly organized and controlled series of migration events which include adhesive interactions and signaling responses is induced for the entrance of immune cells into the target site. In fact, most of our current understanding of the leukocyte recruitment cascade originates from IVM observations of neutrophils, which are also the first and the largest population among the recruited cell types. The classical neutrophil recruitment cascade in most tissues involves the following sequence of steps: tethering, rolling, adhesion, crawling, and transmigration (Kolaczkowska and Kubes, 2013). However, the recruitment cascade of neutrophils in the liver does not involve the P- and E-selectin–dependent tethering and rolling step, highlighting the tissue-specificity of the recruitment paradigm in the liver (Wong et al., 1997; Lee and Kubes, 2008) (Figure 2). Studies using IVM and different murine liver disease models have revealed context-dependent roles of inflammation mediators, chemokines, and adhesion molecules involved in the neutrophil recruitment cascades. In LPS- or *Escherichia coli*-induced sepsis model, the sequestration of neutrophils in liver sinusoids depends on the binding of CD44 and hyaluronan (HA) (McDonald et al., 2008; McDonald et al., 2010; McAvoy et al., 2011). Using a sterile inflammation model induced by localized thermal injury, Kubes et al. found that neutrophils swarm into the injury site in a multistep process strictly controlled by different chemoattractant or adhesion molecules (McDonald et al., 2010). In this model, the interaction of integrin αMβ2 and its endothelial ligand intercellular adhesion molecule-1 (ICAM-1) mediates neutrophil adhesion within liver sinusoids, and the CXCL2–CXCR2 axis controls the intravascular migration of neutrophils toward the border of the injury area, while formyl-peptide released by mitochondria of necrotic cells directs neutrophil chemotaxis to the necrotaxis zone *via* FPR1 (McDonald et al., 2010). The essential role of FPR1 in regulating neutrophil recruitment during liver inflammation was also demonstrated in another sterile injury model induced by hepatic ischemia-reperfusion or laser-irradiation (Honda et al., 2017). Using an unbiased *in vivo* functional screen combined with IVM, dipeptidase-1 (DPEP1) was identified as a novel adhesion receptor mediating neutrophil recruitment in the lungs and liver (Choudhury et al., 2019). Together, these studies provided a paradigm of leukocyte recruitment in the liver. A growing body of evidence has shown that various immune cell types are recruited into the inflamed liver *via* similar recruitment cascades. Although the migration of iNKT cells and CD8^+^ T cells is directed by different chemokine–chemokine receptors, including CXCL16-CXCR6 and CXCL9/CXCL10-CXCR3, their retention in liver sinusoids is dependent on the interaction between β2-integrin and ICAM-1 (Bertolino et al., 2005; Lee and Kubes, 2008; Thomas et al., 2011; McNamara et al., 2017). The adhesion of platelets to sinusoidal KCs relies on the CD44–HA interaction and is necessary for the retention of effector CD8^+^ T cells in liver sinusoids (Guidotti et al., 2015; Malehmir et al., 2019).

The recruitment of certain types of lymphocytes also relies on unique adhesion molecules. For example, the recruitment of CD4^+^ Th1 and Th2 lymphocytes in the liver depends, respectively, on α4-integrin and vascular adhesion protein-1 (VAP-1) (Bonder et al., 2005). A GATA6^+^ peritoneal macrophage population is recruited to the injured tissue during a sterile liver injury. These macrophages adopt a nonvascular route to invade the afflicted tissue directly and repair it rapidly, and this process is dependent on CD44 and alarmin ATP (Wang and Kubes, 2016). The work by Wang et al. revealed another migration pattern of neutrophils in the liver, named “reverse transmigration.” Reverse transmigration is the process by which neutrophils return to the circulation after completing their task at the injury site. It was demonstrated that this process requires the protease cathepsin C, but the identity of the chemoattractant guiding the exit of neutrophils from the injury site and reentry into the circulation remains to be determined (Wang et al., 2017).

The aforementioned findings obtained in IVM studies provided deeper insights into the specific mechanisms of immune cell recruitment during liver inflammation and may offer novel therapeutic targets for the treatment of liver disease.

### Imaging the Immune Cell Functions *In Vivo*

With the advantages of IVM in recording the life of immune cells in their natural environment, the functions of many immune cells have been newly defined across different dimensions. As aforementioned, the processes of bacteria capture, phagocytosis, acidification, and killing through oxidative respiration burst, all performed by phagocytes, can now be observed and evaluated by IVM (Surewaard and Kubes, 2017). In the sterile injury model, neutrophils and GATA6^+^ peritoneal macrophages were recruited to the injury area, where they dismantled dead cells and debris. IVM observations revealed that these cells showed sampling behavior and took up small DNA particles from the nucleus of dead cells. The clearance function of these rapidly recruited phagocytes helps create channels for revascularization and promotes tissue repair during sterile hepatic injury (Wang and Kubes, 2016; Wang et al., 2017).

Neutrophil Extracellular Traps (NETs) are net-like structures comprising DNA–histone complexes, proteases, and granule proteins released by activated neutrophils (Brinkmann et al., 2004; Yipp et al., 2012). NETs released by neutrophils can retain and kill bacteria, preventing their dissemination into the bloodstream and distant organs (Brinkmann et al., 2004; McDonald et al., 2012). NET formation in liver sinusoids can be visualized by intravenous infusion of cell-impermeable DNA dye Sytox Green and fluorescently labeled antibodies specific for histone H2Ax and neutrophil elastase (McDonald et al., 2012). In addition to sepsis, NET formation was also found to take place during sterile inflammation, autoimmunity, coagulation, and cancer (Jorch and Kubes, 2017). NETs may lead to platelet aggregation, intravascular coagulation, and organ damage (Jiménez-Alcázar et al., 2017; McDonald et al., 2017). NET degradation by DNase1 and DNase1-like 3 can significantly reduce vascular occlusions and improve microvascular perfusion, preventing liver damage during sepsis (Jiménez-Alcázar et al., 2017; McDonald et al., 2017). In addition to releasing NETs, neutrophils migrating in the liver sinusoid leave behind retraction fibers that contain cellular contents enclosed in a large vesicular structure termed “migrasome” (Ma et al., 2015; Jiao et al., 2021). Damaged mitochondria are expelled from neutrophils by translocation into migrasomes; this process is conducive to the maintenance of mitochondrial membrane potential and normal respiratory function, which is necessary for sustaining the viability of neutrophils in the liver (Jiao et al., 2021). Importantly, damage-associated molecular patterns (DAMPs), such as formyl-peptide, and mitochondrial DNA released into circulation from damaged mitochondria can trigger the innate immune response (Zhang et al., 2010). How these damaged mitochondria and cytosolic components present in migrasomes affect the immune response in the liver remains to be further evaluated.

### Imaging the Immune Cell Interactions During Inflammation

**FIGURE 3:**
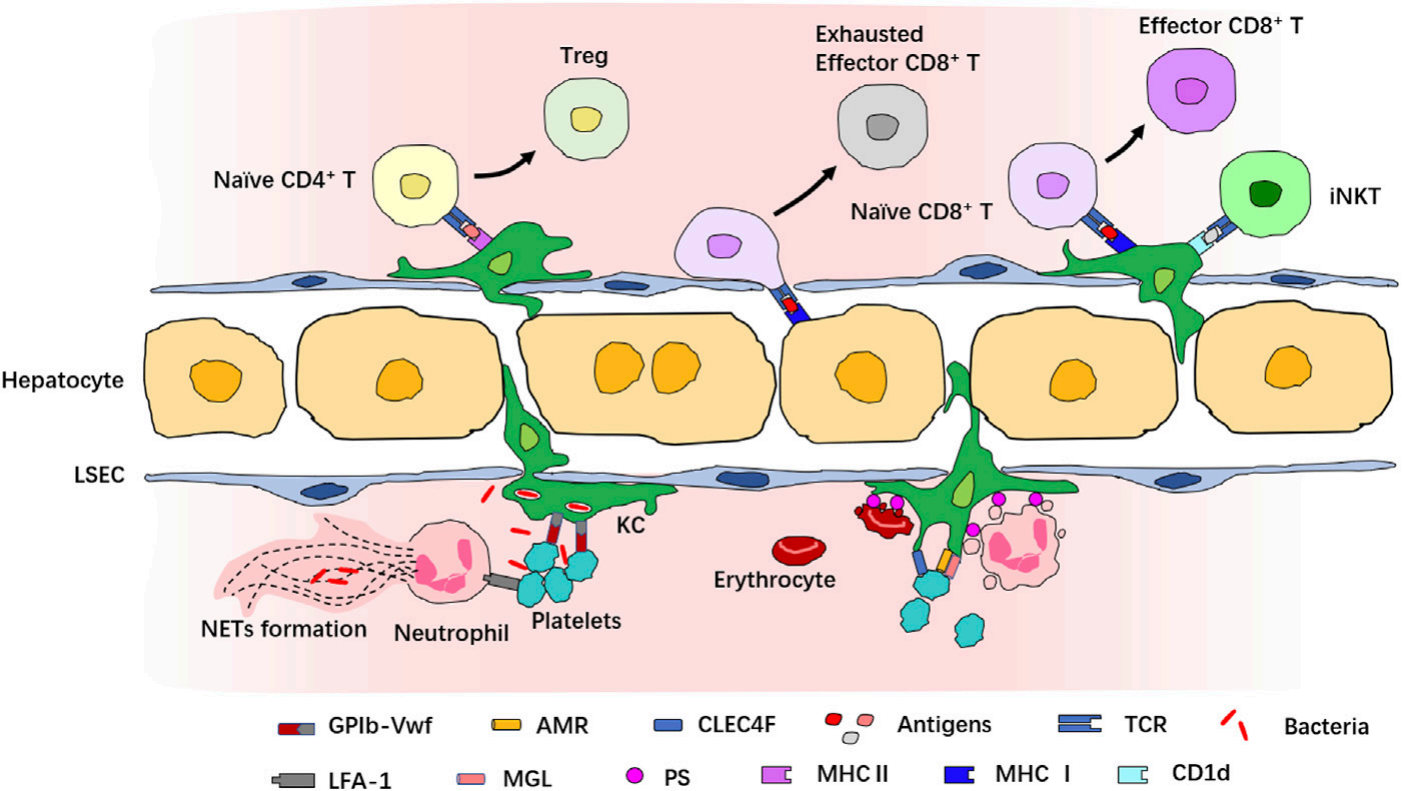
Interaction of immune cells in the liver revealed by IVM. Multiple immune cells interact with each other in both physiological and pathological conditions in the liver. Many cells in the liver can act as antigen-presenting cells to interact with T cells. KCs express both MHCⅠ and MHCⅡ and many costimulatory molecules and present antigens to naive CD4^+^ or CD8^+^ T cells for their activation and priming. The antigen presentation to CD8^+^ T cells by KCs induces full differentiation to effector CD8^+^ T cells, while the antigen presentation to CD4^+^ T cells by KCs induces differentiation into Treg. Hepatocytes also present antigens to CD8^+^ T cells, but this leads to exhaustion of CD8^+^ T cells. KCs activate iNKT cells *via* CD1d. Interaction among KCs, neutrophils, and platelets provides defense against bacteria invasion, and neutrophil–platelet interaction is necessary for NET formation. In addition, KCs also recognize apoptotic cells and interact with senescent and apoptotic erythrocytes, platelets, and neutrophils for the clearance of dying immune cells.

The initiation and resolution of inflammatory response require delicate coordination of the function of immune cells. IVM provides a powerful tool to decode how immune cells interact with each other in the complex architecture and microenvironment to orchestrate inflammatory responses (Figure 3). For example, Surewaard and collaborators had reported that during methicillin-resistant *Staphylococcus aureus* (MRSA) bacteremia, KCs represent an intracellular reservoir for bacteria to avoid attacking by neutrophils (Surewaard et al., 2016). IVM has also unveiled that platelets can cooperate with KCs *via* the interaction of platelet-adhesion receptor GPIb and von Willebrand factor (vWF), helping KCs to capture blood-borne pathogens (Wong et al., 2013). The interaction of KCs with neutrophils and platelets prevents the disseminated bacteria from escaping the KC killing machinery (McCuskey, 1966; Surewaard et al., 2016). Platelets also interact with liver neutrophils during sepsis *via* α_L_β_2_-integrin (LFA-1) which is required for NET formation (Jenne et al., 2011; McDonald et al., 2012).

KCs also interact with neutrophils and platelets in physiological states in the absence of bacterial infection. It is estimated that about 100 billion neutrophils and platelets are generated in humans every day, and similar numbers of apoptotic and senescent neutrophils and platelets must be eliminated from circulation (Quach et al., 2018; Ng et al., 2019). These apoptotic and senescent cells were thought to be cleared mostly by tissue phagocytes in the liver, spleen, and bone marrow in a physiological process called “efferocytosis” (Shi et al., 2001; Furze and Rankin, 2008; Grozovsky et al., 2010; Greenlee-Wacker, 2016). Using IVM, Deppermann and collaborators documented the capture of desialylated platelets by Kupffer cells; this process was mediated by the recognition of aged platelets *via* the binding of the Ashwell–Morwell receptor (AMR) to macrophage galactose lectin (MGL) (Deppermann et al., 2020). A study by Jang and coworkers demonstrated that the desialylated glycans of platelets can be recognized by the C-type lectin receptor (CLEC4F) expressed by KCs (Jiang et al., 2021). Several other studies have reported the contribution of KCs to the clearance of apoptotic neutrophils in the liver (Shi et al., 2001; Hong et al., 2012; Greenlee-Wacker, 2016; Bukong et al., 2018), and the inhibition of KC-mediated phagocytosis results in an increased number of apoptotic neutrophils in the spleen and lungs (Shi et al., 2001). However, this process has not been observed by IVM, which may be due to the limited imaging depth and the scarcity of apoptotic neutrophils in the liver under physiological conditions (Kubes and Jenne, 2018). KCs also clear senescent red blood cells *via* phagocytosis and release iron from heme, maintaining iron homeostasis (Ganz, 2012). This process is termed “erythrophagocytosis” and is mediated by the recognition of erythrocyte aging markers, such as the modification of the erythrocyte membrane protein band 3 and the appearance of phosphatidylserine on the outer leaflet of the plasma membrane (Ravichandran, 2010; Lee et al., 2011).

With the presentation of MHC-I, MHC-II, and costimulatory molecules, LSECs, hepatocytes, and KCs also interact with CD8^+^ T cells to present the antigen, as we have discussed before (You et al., 2008; Bénéchet et al., 2019). The antigen presentation of HBV core protein by KCs leads to full differentiation of HBV-specific naïve CD8^+^ T cells into effector cells. These effector cells form dense, extravascular clusters of immotile cells scattered throughout liver lobules. However, when HBV core protein is presented by hepatocytes, CD8^+^ T cells form loose, intravascular clusters of motile cells around the portal vein and fail to differentiate into effector cells capable of full cytotoxic function (Bénéchet et al., 2019). A study published by Heymann et al. showed that when cognate antigen is presented to CD4^+^ T cells by KCs, activated CD4^+^ T cells differentiate over time into Foxp3^+^ regulatory T cells (Treg) (Heymann et al., 2015b). During *Borrelia burgdorferi* infection, KCs can also present cognate antigens by CD1d and recruit iNKT cells to secrete interferon-γ for host defense (Lee et al., 2010). This evidence partly explains the mechanism of hepatic lymphocyte activation and tolerance. However, the tissue architecture, composition, and activation profiles of immune cells are dramatically altered in pathological states, especially during chronic liver disease; therefore, the process of antigen presentation in an unhealthy liver may require additional evaluation (You et al., 2008; Pose et al., 2021; Triantafyllou et al., 2021).

## Novel IVM Methods to Investigate Dynamic Single-Cell Behavior

Recent advances in microscope technologies, new strains of reporter mice, novel surgical approaches, and broad access to fluorochrome-conjugated antibodies have led to many discoveries using IVM (Pittet et al., 2018). However, many challenges in this area remain to be addressed. New imaging methods are constantly being improved and created to meet the needs of scientific research. Because of the limitations of the optical microscope, fluorescence-based IVM usually requires a trade-off between resolution, speed, signal-to-noise ratio (SNR), and sample health (Laissue et al., 2017). Efforts have been made in the past decade toward the hardware and algorithms of microscopy, such as spinning-disk confocal microscopy (SDCM) (Nakano, 2002), adaptive optics (AO) (Ji et al., 2010), and high-speed two-photon microscopy (Lu et al., 2020). As a result, imaging speed and imaging depth have been greatly improved, and photodamage has been reduced. Inspired by the photomechanical response of the fly eye and the subpixel shift in photography (Llavador et al., 2015), Wu et al. proposed a computational imaging framework combining the digital adaptive optics algorithm with scanning light-field microscopy. This novel platform allows prolonged intravital imaging characterized by high temporal and spatial resolution and low photobleaching (Wu et al., 2021). This system permits 3D IVM observation of the mouse liver for up to 3 h, with a sampling speed of three volumes per second, while providing a resolution high enough to analyze the migrasome (0.5–2 um) dynamics (Ma et al., 2015).

One technical issue faced by IVM users when imaging the liver with chronic disease is autofluorescence. Although this phenomenon can provide real-time information on the morphology and functional properties of the liver (Saif et al., 2020; O'Sullivan et al., 2020), it also hinders the acquisition of high-quality images in the state of chronic liver disease. Davis et al. report a strategy to accommodate autofluorescence for the multicolor visualization of leukocyte behavior in a nonalcoholic fatty liver disease (NAFLD) mouse model. Using the spectral patterns of autofluorescence in the context of NAFLD obtained by progressive emission filter scan (Lambda Scan), the imaging parameters, including narrowed filters, off-peak fluorescence collection, and sequential excitation, were optimized to minimize the background signal. Imaging using this optimized IVM protocol provided details of cell trafficking, recruitment, function, and behavior in fatty liver (Davis et al., 2019). Moon et al. used intravital two-photon microscopy to quantitate lipid droplet accumulation, fibrosis, and disruption of the microvasculature (Moon et al., 2020; Moon et al., 2021). These methods enable studying the inflammatory response and disease progression in NAFLD and can facilitate the development of diagnostic and therapeutic strategies.

The widely used surgical method in IVM usually allows a few hours of observation and thus cannot fulfill the requirement of monitoring long-lasting pathological processes such as the development of chronic liver disease and tumor metastasis. This problem can be addressed by an implanted abdominal imaging window, which allows longitudinal IVM observation for up to 5 weeks and has been used for the visualization of metastasis formation in the liver over 14 days (Ritsma et al., 2012). Deng et al. further developed a drawer-type abdominal window to eliminate the impact of respiration and heartbeat on the quality of imaging (Deng et al., 2021).

The behavior and dynamics of multiple immune cell types engaged in the inflammatory response are highly diverse and are characterized by rapid changes in the motility and morphology of individual cells. Though various software packages provide automatic analysis of high-dimensional imaging data, in most cases, manual curation is still required (Pittet et al., 2018). As a result, the analysis of IVM data is labor-intensive and often induces bias. Recently, Molina-Moreno et al. combined deep learning and machine learning to create a tool for fully automatic analysis of the leukocyte migration (Molina-Moreno et al., 2022). Crainiciuc et al. also provided a method for the visualization of high-dimensional datasets consisting of hundreds of morpho-kinetic parameters describing the dynamics of individual cells; their protocol allows for portraiting of full landscapes of immune cell behavior during inflammation (Crainiciuc et al., 2022). These attempts provide methods for unbiased, high-throughput, and automatic analysis of the dynamics of single-cell behavior. In combination with multicolor IVM (Nakagaki et al., 2019; Dawson et al., 2021), these new approaches offer promising prospects for deciphering inflammatory responses using IVM.

## Discussion

Although the inflammatory response in the liver has been extensively studied using IVM, most of these studies have used artificial models that do not completely reflect the actual liver diseases. In chronic liver diseases, particularly tissue architecture (Michalopoulos and Bhushan, 2021), immune cell heterogeneity, and microenvironment (Tacke and Zimmermann, 2014; Ramachandran et al., 2019; Remmerie et al., 2020; Seidman et al., 2020) are frequently altered, and large parts of current knowledge derived from studying the healthy liver may not be applicable anymore. Thus, imaging cellular behavior in more clinically relevant disease models becomes necessary. Moreover, although IVM is a powerful tool in investigating the dynamic behavior and interaction of immune cells during inflammation, it is difficult to correlate the dynamics and spatial organization of cells with the characteristics of their transcriptome and proteome profiles. The combination of IVM with advanced spatial transcriptomic and proteogenomic techniques may make it possible to obtain comprehensive spatiotemporal information on the inflammatory response and offer broad prospects for the diagnosis and therapy of liver disease.
